# Morbidity After Inguinal Lymph Node Dissections: It Is Time for a Change

**DOI:** 10.1245/s10434-016-5461-3

**Published:** 2016-08-12

**Authors:** Marloes Faut, Rianne M. Heidema, Harald J. Hoekstra, Robert J. van Ginkel, S. Lukas B. Been, Schelto Kruijff, Barbara L. van Leeuwen

**Affiliations:** Department of Surgical Oncology, Department of Surgery, University of Groningen, University Medical Centre Groningen, Groningen, The Netherlands

## Abstract

**Background:**

Inguinal lymph node dissection (ILND) for stage 3 melanoma is accompanied by high wound complication rates. During the past decades, several changes in perioperative care have been instituted to decrease the incidence of these complications. This study aimed to evaluate the effect of these different care protocols on wound complications after ILND.

**Methods:**

A retrospective analysis of prospectively collected data was performed with 240 patients who underwent an ILND in the University Medical Center Groningen between 1989 and 2014. Four groups with different treatment protocols were analyzed: A (≥10 days of bed rest with a Bohler Braun splint), B (10 days of bed rest without a splint), C (5 days of bed rest), and D (1 day of bed rest). The effect of early mobilization, abolishment of the Bohler Braun splint and postural restrictions, and the introduction of prophylactic antibiotics were analyzed.

**Results:**

One or more wound complications occurred in 51.2 % of the patients including wound infection (29.8 %), seroma (21.5 %), wound necrosis (13.6 %), and hematoma (5 %). In consecutive periods, respectively 44.4, 60.3, 44.9 and 55.2 % of the patients experienced wound complications. None of the instituted changes in protocols led to a decrease in wound complications.

**Conclusion:**

Changes in perioperative care protocols did not affect the rate of wound complications. Perhaps a change in the surgical procedure itself can lead to the necessary reduction of wound complications after ILND.

For stage 3 melanoma patients, regional lymph node (LN) dissection is the standard surgical therapy after a positive sentinel LN biopsy [completion LN dissection (CLND)] or clinically palpable LNs [therapeutic LN dissection (TLND)]. Unfortunately, over the decades, LN dissection of the inguinal region (ILND) has been associated with high rates of wound complications, with rates of wound infection (WI), seroma, and wound necrosis as high as 46 %.^1^–^4^

The literature on predictive factors for postoperative early wound complications is scarce and sometimes contradictory. Factors such as age, body mass index (BMI), smoking, comorbidities, the influence of palpable nodal disease, and the duration of postoperative bed rest all have been analyzed extensively.^2^,^4^–^8^

In the early 1970s, it was hypothesized that strict bed rest with the use of a Bohler Braun splint during the postoperative period would lead to edema reduction and a decreased tension on the wound, with a potential lower incidence of wound complications. However, treatment protocols regarding the duration of postoperative bed rest and the use of a Bohler Braun splint have changed over time at our institution. Postoperative bed rest duration was reduced to increase cost effectiveness and to decrease the incidence of venous thrombotic events. Subsequently, from 2004 onward at our institution, prophylactic perioperative antibiotics have been introduced, aimed at decreasing infection rates in CLNDs.

This retrospective review of prospectively collected data aimed to evaluate the different peri- and postoperative protocol adjustments over the years and to evaluate their influence on wound complications in a tertiary melanoma referral center.

## Materials and Methods

The University Medical center Groningen (UMCG) is a melanoma referral center. Patients who underwent an ILND for stage 3 melanoma between 1989 and 2014 were included in a database. The surgical procedure was either a superficial LN dissection or a combined superficial and deep LN dissection. Data were collected concerning patient characteristics, tumor characteristics, the operation procedure, and the postoperative period.

A single elliptical incision was used over the femoral triangle, and skin was resected together with the superficial specimen. The superficial specimen consisted of fat and subcutaneous tissue between Scarpa’s fascia and the muscular layer. The saphenous vein was always transected. For the combined dissections, the inguinal ligament was divided for entrance to the deep pelvis. The deep dissection was performed along the external iliac artery. A sartorius flap was used, and two vacuum drains were left behind.^1^,^2^

During the entire study period, the same disinfectant, Chloride Hexidine, was used. Since 2007, a door movement protocol has been instituted, and door movements during surgery have been strictly limited to necessary door movements. The temperature in the operating room is always kept at 18 °C.

Histologic examination of sentinel LNs was performed with hematoxylin-eosin (H&E) staining. If H&E staining was positive, a combined superficial and deep LN dissection was performed. In case of a negative H&E staining, immunohistochemical (IHC) staining for the protein S100 and HMB45 was performed. If IHC staining was positive, the patient was considered solely for a superficial LN dissection from 2002 onward. For patients with positive IHC staining who underwent a CLND, an additional deep LN dissection was performed if additional positive LNs were found in the dissected specimen. From 2004 onward, prophylactic perioperative antibiotics (1000 mg of intravenous cefazolin) were administered for all patients scheduled for a CLND. Patients with more than three involved nodes, a LN metastasis larger than 4 cm, or extra capsular extension of tumor growth received adjuvant radiotherapy (48 Gy in 20 fractions given during a maximum of 30 days) with or without trial participation.^9^

Wound complication was defined as a wound complication within 30 days after the ILND. The wound complications were divided into four categories: 1 (wound infection requiring antibiotics, surgical intervention, or both), 2 (wound necrosis inducing secondary wound healing and/or requiring surgical intervention), 3 (seroma formation requiring needle aspiration), and 4 (hematoma requiring surgical intervention). Seroma was defined as a fluctuating swelling in the inguinal area, and hematoma was defined as a (fluctuating) swelling in the inguinal area caused by a bleeding. An inguinal hernia requiring surgical correction and erysipelas, urine tract infection, partial neuropraxia, urinary retention, pulmonary embolism, or delirium was defined as “other complication.”

Four perioperative care protocols were used over the years: A (≥10 days of bed rest with a Bohler Braun splint), B (10 days of bed rest without a splint), C (5 days of bed rest), and D (1 day of bed rest). These protocols correspond respectively with the periods 1989**–**2000, 2001–2005, 2006–2011, and 2012–2014. The vacuum wound drains were removed after a minimum of 7 days and if production was less than 20 ml per 24 h. Patients were prescribed support stockings for the first 6 months, and low-molecular-weight heparin was administered subcutaneously (2850 IU of Fraxiparine) during immobilization.

Statistical analysis was performed with IBM SPSS version 22.0 (IBM, Inc., Chicago, IL, USA). For continuous variables, one-way analysis of variance (ANOVA) or the Kruskal–Wallis test was used. Differences between nominal variables were analyzed using the chi-square test. Variables with a 20 % significance level in the univariate logistic regression were entered into the multivariate regression. Variables with a *p* value lower than 0.05 in the multivariate logistic regression were identified as significant factors associated with wound complications. The following variables were analyzed for their potential association with wound complications according to the literature: age, gender, smoking, BMI (<25 vs. 25**–**30 vs. >30 kg/m^2^), comorbidity (diabetes mellitus, pulmonary disease, and cardiovascular disease), CLND versus TLND, and nodal yield. The expected nodal yield was 8 to 10 LNs for a superficial dissection and 4 to 6 LNs for the deep dissection.

The nodal yield was entered as a categorical variable (0–16 LNs, 17–22 LNs, and >22 LNs). The patients with a superficial dissection only were not included in the nodal yield analysis. The details of the operation included operation time (≤130 vs. >130 min) and superficial versus combined dissection. Operation time was defined as the time from skin incision until skin closure. Melanoma-specific survival (MSS) and disease-free survival (DFS) were calculated with a Kaplan–Meier analysis. Survival was compared between patients with and patients without a wound complication and between patients with and patients without a WI. The primary end point was the occurrence of early (≤30 days) postoperative wound complications in patients after ILND. Institutional review board approval was achieved, and the study was conducted according to the declaration of Helsinki.

## Results

The study included 244 ILNDs for 239 patients (114 males and 125 females) with a median age of 56 years (range, 5–91 years). The general clinicopathologic characteristics are summarized in Table 1. The majority of the patients (95 %) underwent a combined superficial and deep ILND. Two patients underwent a concurrent bilateral ILND. Three patients underwent a bilateral superficial and deep ILND in two separate surgical procedures.

**Table 1:** Patient characteristics

Characteristic	*n*	%	Median	Range
Gender				
Male	114	47.7		
Female	125	52.3		
Age (years)^a^			56	5–91
Location of primary tumor				
Trunk	25	10.5		
Genital area	4	1.7		
Thigh	70	29.3		
Lower leg	73	30.5		
Foot	44	18.4		
Mucosal	1	0.4		
Unknown primary	22	9.2		
Histology of primary				
Superficial spreading	96	39.7		
Nodular melanoma	49	20.2		
Acrolentiginous	23	9.5		
Unknown primary tumor	22	9.2		
Other^b^	17	7.0		
Breslow thickness (mm)			2.5	0.6–27
T1 (≤1.00)	19	7.9		
T2 (1.01–2.00)	62	25.9		
T3 (2.01–4.0)	81	33.9		
T4 (>4.0)	53	22.2		
Unknown primary	22	9.2		
Ulceration				
Yes	89	37.2		
No	116	48.5		
Unknown primary	22	9.2		
Comorbidity				
BMI >25	137	57.3		
Smoking, current	64	26.8		
≥1 comorbidity^c^	83	34.7		
Diabetes mellitus	18	7.5		
Cardiac disease	46	19.2		
Vascular disease	38	15.9		
Pulmonary disease	19	7.9		
Indication^d^				
Micrometastasis	75	31		
Macrometastasis	167	69		
Dissection type				
Superficial and deep	230	95		
Superficial	12	5		
Radiotherapy <3 months^e^				
Yes	65	26.9		
No	168	69.4		
Unknown	9	3.7		

*BMI* body mass index

^a^Age at lymph node dissection

^b^Other is defined as verrucus, spitzoid, epitheloid, desmoplastic melanoma and lentigo maligna melanoma

^c^Patients with ≥1comorbidity including cardiac and/or vascular and/or pulmonary disease and/or diabetes mellitus

^d^Macrometastasis is defined as a palpable inguinal lymph node; micrometastasis is defined as tumor load in the sentinel node

^e^Radiotherapy started within 3 months after lymph node dissection

Overall, one or more wound complications occurred after 124 (51.2 %) of the ILNDs. Wound infection was the most frequent complication (*n* = 72, 29.8 %), followed by seroma (*n* = 52, 21.5 %), wound necrosis (*n* = 33, 13.6 %), and hematoma (*n* = 12, 5 %) . Antibiotics were prescribed for 72 patients (29.8 %) postoperatively. Of these patients, 72 experienced a WI and 4 had another infection such as a urine tract infection. Erysipelas was encountered during the 30-day postoperative period by 19 patients (7.9 %). Surgical intervention was performed for seroma, hematoma, or an abscess for 49 patients (20.2 %). Two patients (0.8 %) experienced postoperative bleeding, which required reexploration. One patient died of a cardiac arrest during hospitalization. Other complications occurred for 48 patients, the majority of which were erysipelas, urine retention, urine tract infection, and inguinal hernia.

Three patients (1.2 %) experienced a pulmonary embolism within 3 months after surgery despite their use of prophylactic subcutaneous heparin. No deep venous thrombosis was seen. Two of these patients were overweight. The durations of bed rest for the three patients were respectively 5, 8, and 10 days.

We observed no thromboembolic events in the group with a short period of bed rest. More than half of the patients (57.3 %) had a BMI higher than 25 kg/m^2^, and 34.7 % of the patients had more than one comorbidity. Postoperative radiotherapy was performed for 65 patients (26.9 %), received within 3 months after the ILND. Eight patients (3.3 %) missed adjuvant radiotherapy due to postoperative wound complications.

The differences between the four perioperative care protocols are presented in Table 2. After the introduction of SLNB, TLND was performed less frequently during the different periods (*p* = 0.020).

**Table 2:** Comparison of clinicopathologic characteristics between different care protocols (*n* = 239)^a^

Characteristic	A (1989–2000)	%	B (2001–2005)	%	C (2006–2011)	%	D (2012–2014)	%	*p* value^a^
	(*n* = 63)		(*n* = 78)		(*n* = 69)		(*n* = 29)		
Median age: years (range)	55 (20–80)		54.5 (22–86)		59 (5–91)		59 (20–74)		0.453
≤55	33	52.4	41	52.6	29	42	12	41.4	
>55	30	47.6	37	47.4	40	58	17	58.6	
Gender									0.687
Male	30	47.6	40	51.3	29	42	15	51.7	
Female	33	52.4	38	48.7	40	58	14	48.3	
Smoking, current									0.347
No	43	68.3	57	73.1	50	72.5	25	86.2	
Yes	20	31.7	21	26.9	19	27.5	4	13.8	
Histology of primary tumor									**<0.001**
Superficial spreading	14	22.2	29	37.7	40	58	12	41.4	
Nodular melanoma	12	19.0	11	14.3	16	23.2	8	27.6	
Acrolentigenous	8	12.7	10	13.0	2	2.9	3	10.3	
Unknown primary tumor	7	11.1	8	10.3	5	7.2	2	6.9	
Other^b^	3	4.8	6	7.8	4	5.8	4	13.8	
Median BMI: kg/m^2^ (range)	26 (18.7–39.1)		26 (20.2–39.8)		25.1 (13.5–53.9)		26 (20.2–36.8)		0.151
<25	25	41.0	27	36.0	33	48.5	11	37.9	
25–30	30	49.2	39	52.0	24	35.3	10	34.5	
>30	6	9.8	9	12.0	11	16.2	8	27.6	
Diabetes mellitus									0.347
No	60	95.2	74	94.9	61	88.4	26	89.7	
Yes	3	4.8	4	5.1	8	11.6	3	10.3	
≥1 Comorbidity^c^									0.332
No	42	66.7	55	70.5	44	63.8	15	51.7	
Yes	21	33.3	23	29.5	25	36.2	14	48.3	
Indication									**0.020**
Micrometastasis	18	28.6	17	21.8	25	36.2	15	51.7	
Macrometastasis	45	71.4	61	78.2	44	63.8	14	48.3	
Median OR time: min (range)	135 (40–285)		141 (68–254)		125 (50–248)		130 (70–195)		0.168
≤130	31	49.2	30	38.5	39	56.5	15	51.7	
>130	32	50.8	48	61.5	30	43.5	14	48.3	
Median postoperative hospital stay: days (range)	14 (7–34)		13 (8–45)		8 (5–47)		6 (4–14)		**<0.001**
Radiotherapy									0.823
No	42	72.4	55	73.3	46	67.6	22	75.9	
Yes	16	27.6	20	26.7	22	32.4	7	24.1	
≥1 Wound complication									0.173
No	35	55.6	31	39.7	38	55.1	13	44.8	
Yes	28	44.4	47	60.3	31	44.9	16	55.2	
Wound infection	16	24.5	22	28.2	22	31.9	12	41.4	0.448
Seroma	13	20.6	18	23.1	12	17.4	9	31	0.500
Hematoma	0	0	5	6.4	2	2.9	4	13.8	
Necrosis	6	9.5	20	25.6	4	5.8	3	10.3	
Complication grades 1–2^d^	25	71.4	50	76.9	27	67.5	17	60.7	0.333
Complication grade 3	10	28.6	15	23.1	13	32.5	11	39.3	0.093
≥1 Other complication									0.613
No	51	81	62	79.5	58	84.1	21	72.4	
Yes	12	19	16	20.5	11	15.9	8	27.6	
Total nodal yield									**0.003**
0–16	34	73.9	46	64.8	25	43.1	11	42.3	
17–22	10	21.7	17	23.9	18	31	6	23.1	
>22	2	4.3	8	11.3	15	25.9	9	34.6	

*BMI* body mass index, *OR* operating room

Group A had a Bohler Braun splint and ≥7 days of bed rest. Group B, C, and D had the leg elevated and no Bohler Braun splint. Group B had ≥7 days of bed rest. Group C had 5 or 6 days of bed rest. Group 4 had 1–4 days of bed rest

^a^ *p* Values <0.05 are in bold

^b^Other is defined as verrucus, spitzoid, epitheloid, desmoplastic melanoma, and lentigo maligna melanoma

^c^Patients with ≥1 comorbidity included those with cardiac and/or vascular and/or pulmonary disease and/or diabetes mellitus

^d^Grades are for surgical complications: 1–2 mild to moderate (observation or antibiotics), 3 severe (surgical intervention)

Prophylactic antibiotics in the CLND group had no impact on the incidence of wound complications (*p* = 0.143) nor on the incidence of postoperative WIs (*p* = 0.830). The institution of the door movement protocol in 2007 did not lead to a reduction in WIs (*p* = 0.180). The occurrence of wound complications did not differ between the different perioperative care protocol groups (*p* = 0.173) nor between CLND and TLND (*p* = 0.499). The extent of the dissection (superficial + deep vs superficial) did not influence the incidence of wound complications (*p* = 0.496). Operating time did not differ significantly between CLND and TLND (*p* = 0.187).

Multivariate analysis showed increasing age to be associated with the occurrence of wound complications [odds ratio (OR), 1.03 per year; *p* = 0.035], as shown in Table 3. The analysis showed a BMI higher than 30 kg/m^2^ to be independently associated with WI, as shown in Table 4 (OR, 2.93; *p* = 0.013). Data on recurrence and survival were available for 182 patients, and 57 patients were lost to follow-up. The median MSS was worse for the patients with a wound complication (27.4 months; range, 18.9–35.9 months) than for the patients without a wound complication (88.8 months; *p* = 0.002). The median DFS was worse for the patients with a wound complication (10.5 months; range, 6.7–14.4 months vs. 30.6 months; range, 13.7–47.5 months; *p* = 0.001). The median DFS for the patients with a WI (10.5 months; range, 6.2–14.9 months) also was worse than for the patients without a WI (21.9 months; range, 11.5–32.3 months) (*p* = 0.006).

**Table 3:** Uni- and multivariate analyses of characteristics associated with ≥1 postoperative complication (*n* = 124)

Characteristic	≥1 Complications			Multivariate OR, *p* value (95 % CI)
		%	Univariate *p* value^a^	
Median age: years (range)	58.5 (22–91)		**<0.001**	1.03, **0.035** (1.00–1.05)
<55			1.00	
>55			2.16, **0.003**	
Gender				
Male	65/116	56	0.153	1.71, 0.072 (0.95–3.14)
Female	59/126	46.8		
Histology of primary tumor			0.415	
Superficial spreading	47	37.9		
Nodular	25	20.2		
Acrolentigenous	12	9.7		
Unknown primary tumor	11	8.9		
Other	6	4.8		
BMI (kg/m^2^)			**0.018**	
<25	40/98	40.8		1.00, 0.242
25–30	61/104	58.7		1.63, 0.136 (0.86–3.10)
>30	20/34	58.8		1.78, 0.236 (0.76–3.00)
Smoking				
No	89/176	50.6		
Yes	35/66	53	0.733	
≥1 Comorbidity				
No	69/159	43.4		
Yes	55/83	66.3	**0.001**	1.51, 0.236 (0.76–3.00)
Operative time (min)				
≤130	52/117	44.4		
>130	72/125	57.6	**0.041**	1.61, 0.107 (0.90–2.88)
OR year			0.667	
1989–2000	28/63	44.4		
2001–2005	48/80	60		
2006–2011	31/69	44.9		
2012–2014	17/30	56.7		
Bohler Braun splint				
Yes	28/63	44.4	0.211	
No	96/179	53.6		
Bed in linido				
Yes	66/120	54.1	0.246	
No	58/122	47.5		
Dissection type			0.499	
Superficial	5/12	41.7		
Superficial + deep	119/230	51.7		
Indication			0.499	
Micrometastasis	36/75	48		
Macrometastasis	88/167	52.7		
Total nodal yield			0.180	
0–16	60/127	47.2		1.00, 0.767
17–22	28/51	54.9		1.00, 0.998 (0.49–2.03)
>22	20/34	58.8		1.35, 0.482 (0.59–3.07)

*OR* odds ratio, *CI* confidence interval, *BMI* body mass index, *OR* operating room

^a^ *p* Values <0.05 are in bold. All variables with a significance level of *p* < 0.2 in the univariate analysis were entered into the multivariate analysis

**Table 4:** Uni- and multivariate analyses of characteristics associated with wound infection (*n* = 72)

Characteristic	Wound infection			Multivariate OR, *p* value (95 % CI)
		%	Univariate *p* value^a^	
Median age: years (range)	58, (29–91)		**0.031**	1.02, 0.171 (0.99–1.04)
Gender			0.207	
Male	39/116	33.6		
Female	33/126	26.2		
Histology of primary tumor			0.219	
Superficial spreading	29	40.3		
Nodular melanoma	17	23.6		
Acrolentigenous	9	12.5		
Unknown primary	4	5.6		
Other	4	5.6		
BMI (kg/m^2^)			**0.004**	
<25	21/98	21.4		1.00, **0.043**
25–30	35/104	33.7		1.60, 0.158 (0.83–3.06)
>30	16/34	47.1		2.93, **0.013** (1.26–6.85)
Smoking			0.252	
No	56/176	31.8		
Yes	16/66	24.2		
≥1 Comorbidity			**0.015**	
No	39159	24.5		
Yes	33/83	39.8		1.45, 0.268 (0.75–2.80)
Operative time (min)				
≤130	31/117	26.5		
>130	41/125	32.8	0.284	
OR year			0.785	
1989–2000	16/63	25.4		
2001–2005	22/80	27.5		
2006–2011	22/69	31.9		
2012–2014	12/30	40		
Bohler Braun splint			0.380	
Yes	16/63	25.4		
No	56/179	31.3		
Bed in linido			0.228	
Yes	40/120	33.3		
No	32/122	26.2		
Dissection type			0.360	
Superficial	5/12	41.7		
Superficial + deep	67/230	29.1		
Indication			0.263	
Micrometastasis	26/75	34.7		
Macrometastasis	46/167	27.5		
Total nodal yield			0.291	
0–16	35/127	27.6		
17–22	21/51	41.2		
>22	11/34	32.4		

*BMI* body mass index, *OR* operating room

^a^ *p* values <0.05 are in bold. All variables with a significance level of *p* < 0.2 in the univariate analysis were entered into the multivariate analysis

## Discussion

In this retrospective observational study, the different adjustments of treatment protocols were studied over the years with regard to their influence on the occurrence of wound complications after an ILND. Reducing postoperative bed rest did not influence the overall occurrence of wound complications in this study.

Stuiver et al.^8^ in a study of 145 cases, found age to be the only predictor for a wound complication. Reducing the postoperative bed rest also did not influence the wound complication rate in their study. The other studied variables showed great similarity with those in the current study cohort. A difference however, was the variety of surgical techniques used by Stuiver et al.^8^ Due to the consistency of the surgical procedure at our center, we are unable to determine whether changing this procedure would have led to a decrease in postoperative wound complications. In accordance with the results of Stuiver et al.^8^ an earlier analysis of a smaller cohort (*n* = 204) also did not show an association between early mobilization and wound complications.^10^

Reduction of bed rest significantly decreased the hospital stay in the current study, as expected. This is in accordance with the literature.^11^ It seems safe to abolish the 1-week strict bed rest. After the reduction of strict bed rest, the use of a Bohler Braun splint and the postural restrictions (bed positioned with elevated legs) were abolished from 2001 onward. Neither of these changes influenced the occurrence of wound complications. After the postoperative changes, a preoperative change was made with the introduction of prophylactic antibiotics before CLND from 2004 onward. This did not result in a reduction of the wound complication rates or a reduced WI rate.

The addition of IHC staining over time has led to an earlier detection of occult microscopic metastatic tumor cells.^12^ The relative overall increase in CLNDs might also be explained by this change in guidelines. Our finding that a CLND versus a TLND does not influence the incidence of wound complications is in contradiction with findings in the literature. Faries et al.^13^ stated that CLND was accompanied with less morbidity than TLND. This difference in morbidity could be explained by the difference in extent of soft tissue dissection to clear the LN basin. Our results showed no difference in the incidence of wound complications between superficial and combined dissections. Due to the limited numbers of patients in the superficial group (*n* = 12), no definitive conclusion can be drawn from these results.

The institution of a door movement protocol in 2007 did not lead to a reduction of WIs. Knobben et al.^14^ performed a prospective trial at our center, in which multiple behavioral changes (e.g., restriction of door movement) led to a significant reduction of WIs. Since the introduction of the door movement protocol, no major changes have occurred in treatment protocols. Several patient-specific factors, such as BMI and age, do negatively influence the occurrence of wound complications, although these are not subject to intervention.

The multivariate analysis showed no association between the different patient characteristics, the different protocols, or the occurrence of wound complications. The wound complication rate of 51.2 % found in this study is in accordance with rates found in the recent literature.^4^,^5^,^10^,^15^ However, when postoperative WIs were specifically investigated, BMI was associated with their occurrence. Our data show a WI rate of 29.8 % compared with 45 % by Stuiver et al. using the same definition.^8^ The majority of the patients (57.3 %) in the current study were overweight. A trend toward an increase in BMI was found in the most recent population with stage 3 melanoma. More than 25 % of the patients in group D had a BMI higher than 30 kg/m^2^ compared with approximately 10 % in the remaining groups. The finding that BMI adds to the risk for the development of WI is supported by the literature.^4^,^6^,^10^,^11^,^16^ The heterogeneity in reported wound complication rates can probably be explained by variations in definitions used for WI worldwide.^17^

Due to its retrospective nature, the wound complication rate in this study may be lower than found in prospective studies. As we know, immobilization increases the risk of thromboembolic events, particularly in a population with a high BMI.^8^–^22^ Especially in these high-risk patients, early mobilization is of utmost importance. Our data support this because we observed no thromboembolic events in the group with a short period of bed rest. The significant increase in nodal yield over the years is indicative of the success of standardized surgical and pathologic procedures.

Several authors have reported the use of incisional negative pressure wound therapy (INPWT) to prevent WIs at surgical sites, including inguinal incisions.^23^ Prospective randomized studies concerning the application and cost effectiveness of INPWT in oncologic procedures such as an ILND are scarce. Nevertheless, there might be a role for INPWT after ILND in the future.

In conclusion, during the past decades, several adjustments have been made in the treatment protocols for patients undergoing an ILND. To date, none of these adjustments have led to a substantial reduction in wound complications at the UMCG. However, we have learned that bed rest and, with that, hospital admission can be reduced. In general, we can state that when inguinal lymphadenectomies are performed for patients with stage 3 melanoma at the UMCG, the occurrence of wound complications for about 50 % of the patients cannot be avoided to date.

Managing the postoperative patient after ILND with the aim to prevent wound complications remains a challenge, especially taking into account the negative influence of older age and obesity on the occurrence of wound complications and their expected increase in the future.^24^ An ILND is a potentially curative surgical procedure, and wound complications can hinder the most adequate treatment because radiotherapy can be postponed or even abandoned due to wound problems. Furthermore, our results show that both DFS and MSS are significantly worse when a WI occurs. Preventing wound complications is of the essence.

The inability to reduce the incidence of wound complications over the years calls for drastic measures. The most consistent variable over the years has been the surgical procedure itself. Replacement of the large inguinal incision by three smaller incisions away from the inguinal skinfold might offer a solution via a minimally invasive technique, namely, videoscopic inguinofemoral lymphadenectomy. This procedure is accompanied by a lower complication rate in other centers and has a comparable oncologic outcome.^25^–^27^ Because all other adjustments in perioperative care and management have failed, this procedure might be a promising method for reducing wound complications after ILNDs. The authors have started a trial to study the effect of videoscopic inguinofemoral lymphadenectomy on postoperative complications, lymphedema, and quality of life. The first results are expected by early 2017.
